# The regulation effect of WNT-RAS signaling in hypothalamic paraventricular nucleus on renal fibrosis

**DOI:** 10.1007/s40620-019-00637-8

**Published:** 2019-08-07

**Authors:** Guang Zhou, Jiawen Li, Tao Zeng, Peiliang Yang, Aiqing Li

**Affiliations:** State Key Laboratory of Organ Failure Research, Division of Nephrology, Nanfang Hospital, Southern Medical University, Guangzhou, 510515 China

**Keywords:** Renal fibrosis, Wnt/β-catenin signaling, RAS, PVN

## Abstract

**Background:**

Abnormal activation of wnt/β-catenin signaling and renin-angiotensin system is known to play a vital role in the development and progression of CKD. We hypothesized that abnormal expression of central wnt/β-catenin signaling and renin-angiotensin system (WNT-RAS signaling) was tightly involved in CKD.

**Methods:**

We established sham-operated and 5/6 nephrectomized (5/6 NX) rat model and blocked the central wnt signaling by intracerebroventricular injection of adeno-associated virus vector which can overexpress target gene DKK1. The central and renal expression level of wnt/β-catenin signaling and RAS and renal injury were assessed.

**Results:**

The expression levels of the main wnt/β-catenin signaling components in both brain and kidney of 5/6NX rats, such as wnt3a and active-β-catenin, were elevated obviously and the up-regulation were inhibited by central blockade of the wnt signaling. Furthermore, the expression of the major components of RAS in both brain and kidney in 5/6NX rats, such as angiotensinogen (AGT), angiotensin converting enzyme (ACE-1), angiotensin II AT1-receptor (AT1R), was significantly up-regulated and the up-regulated expression was inhibited by central blockade of the wnt singling. Notably, central blockade of the wnt signaling improved renal function as indicated by decreased serum creatinine and 24 h urinary protein, and attenuated interstitial fibrosis as indicated by reduced Sirius red staining and expression of Fibronectin, Collagen-I and α-SMA.

**Conclusion:**

These data suggest that the central WNT-RAS signaling plays a significant role in the development and progression of CKD.

## Introduction

Wnt/β-catenin signaling plays an essential role in embryonic development, tissue homeostasis as well as organic injury and repairment [1–3]. Its abnormal activation is associated with multiple diseases, such as hereditary diseases, cancer, and kidney diseases [3–6]. Accumulating evidence indicates that wnt/β-catenin signaling is activated in a variety of types of renal diseases and contributes to the development and progression of renal fibrosis [5, 7, 8]. Recent studies have uncovered that the components of RAS are directly regulated by wnt/β-catenin in several kidney disease models [9, 10]. RAS genes contain putative T cell factor (TCF)/lymphoid enhancer factor (LEF)-binding sites in their promoter regions, and β-catenin induces the binding of LEF-1 to these sites in kidney tubular cells. Over-activity of either β-catenin or different wnt ligands induces the expression of all RAS genes [11–14].

It is also well acknowledged that activation of intrarenal renin–angiotensin system (RAS) plays a pivotal role in the pathogenesis of chronic kidney disease (CKD), and the antagonists of RAS, such as angiotensin converting enzyme inhibitors (ACEIs) and angiotensin receptor blockers (ARBs), are widely used in the treatment of CKD [15, 16]. The abnormal regulation of RAS, including the intrarenal and cerebral RAS, is tightly associated with the onset and progression of renal injury [17]. It is reported that blocking cerebral RAS by intracerebroventricular injection (ICV) of losartan down-regulates intrarenal RAS expression in CKD rat model. Simultaneously, central blockade of cerebral RAS obviously lowers central and renal sympathetic nerve activity, alleviates renal fibrosis, and delays the progression of CKD [17–19].

In recent years, Reno-Cerebral communication mechanism has been wildly studied and well established. The related mechanisms include the following: (1) Sympathetic afferent and efferent nerves serve as a bridge to connect kidney and brain, and (2) Signaling molecules, such as the components of RAS, directly bind to receptors distributed in the nucleus which lacks blood–brain barrier and result in cascade reactions. It has been well recognized that the hypothalamic paraventricular nucleus (PVN) is a vital integrative nucleus that expresses RAS and can markedly influence blood pressure and sympathetic nerve activity [17, 19]. There has been a considerable amount of research done on the functions of wnt/β-catenin signaling and RAS in renal fibrosis in CKD. However, several significant issues remain to be resolved, such as, whether there is communication between wnt/β-catenin signaling and RAS in brain in CKD? Whether there is communication between cerebral WNT-RAS signaling and intrarenal WNT-RAS signaling in CKD? What is the role of cerebral WNT-RAS signaling on CKD?

Thus, we hypothesized that abnormal activation of cerebral WNT-RAS signaling contributes to the development and progression of CKD. To test our hypothesis, 5/6 nephrectomy (5/6 NX) rat model was established and the central wnt signaling was blocked by ICV AAV-DKK1.

## Materials and methods

### Animals

Five-week-old male Sprague–Dawley (SD) rats purchased from the Nanfang Hospital Animal Experiment Centre were maintained in a pathogen-free facility under temperature- and light-controlled conditions (24 ± 2 °C, 12 h dark/light cycle) and 55 ± 5% humidity for at least 1 week prior to the experiments. All animal experiments were approved by the Animals Experiment Ethics Committee of Southern Medical University, Guangzhou, China.

### Treatment

Five-sixths nephrectomy and sham operation were performed at 6 weeks of age as previously described [17]. In brief, the rats were anesthetized by sodium pentobarbital (40 mg/kg, i.p.) and upper and lower poles of left kidney were excised followed by removing right kidney 1 week later. Six weeks after operation, the rats were then randomly assigned to three groups matched for 24 h urine protein, including: (1) sham group: Intracerebroventricular injection (ICV) of AAV Vector (without target gene); (2) ICV vector+5/6NX group (3) ICV DKK1+5/6NX group: The AAV-DKK1 vectors (Shanghai Genechem Co. Ltd.) were injected into lateral ventricles under the anesthetized at the dose of 6 × 10^10^ vg. The process of intracerebroventricular injection was performed as we previously described [19].

### Double-staining immunofluorescence

Paraffin-embedded tissue samples were prepared as described [20]. The brain slides were treated with primary antibodies against GFP (Abcam, ab1218) and DKK1 (Abcam, ab109416) overnight at 4 °C. Then the slides were treated with secondary fluorescent antibodies 1 hour at room temperature and viewed under a microscope (Zeiss, Axio Imager Z2).

### Histology and immunohistochemistry

Paraffin-embedded Sections (2 μm) of kidney were stained with periodic acid-Schiff (PAS), and Sirius red by routine protocol [19]. Immunohistochemistry was performed by routine method [20]. Primary antibodies include wnt3a (Abcam, ab28472), active-β-catenin (Millipore, 05-665), DKK1 (Abcam, 109416), AGT (Abcam, ab213705), ACE-1 (Abcam, ab222739), AT1 (Abcam, ab18801).

### Western blot

Western blot was performed as previously described [17] using primary antibodies including active-β-catenin (Millipore, 05-665), AGT (Abcam, ab213705), ACE-1 (Abcam, ab222739), AT1 (Abcam, ab18801), FN (Abcam, ab2413), collagen-I (Boster, BA0325), and α-SMA (Sigma, A5228).

### Measurement of systolic blood pressure and renal function

Systolic blood pressure was monitored by the tail-cuff method. Serum creatinine concentration was measured with an automated chemistry analyzer (AU480; Beckman Coulter). Twenty-four hour urine was collected by metabolic cage and 24 h urinary protein was measured using a Coomassie brilliant blue assay (Beyotime, ST030).

### Statistical analyses

All statistical data were expressed by mean ± SEM. The software of IBM SPSS statistics 23 was implied to analyze the data. Data comparison between groups was performed by one-way ANOVA and Student–Newman–Keuls (SNK) test. P < 0.05 was considered statistically significant.

## Results

### Adeno-associated virus vector successfully mediated over-expression of target gene DKK1 in PVN

**Fig. 1:**
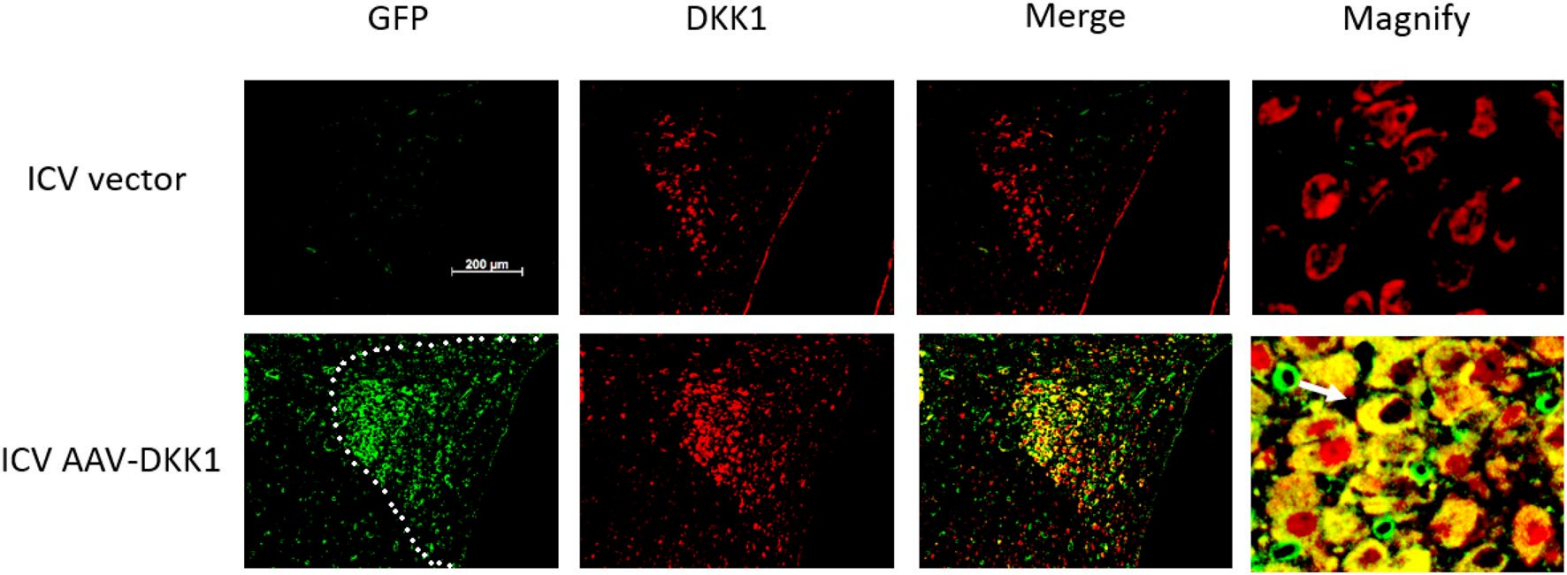
Adeno-associated virus vector successfully over-expressed target gene DKK1 in PVN. Co-localization of GFP and DKK1 in PVN was performed by double staining with the antibody of against GFP (green) and DKK1 (red). The white arrow indicate positive cells with co-localization of GFP and DKK1 (yellow). The white dotted area indicate PVN (color figure online)

To investigate the function of central WNT-RAS signaling in development and progression of renal disease, this study targeted to block the wnt signaling by AAV-DKK1 which can over-express DKK1 gene in PVN in CKD rats. Double-staining immunofluorescence showed that the marker gene green fluorescent protein (GFP) only expressed in ICV AAV-DKK1 group, and DKK1 concentration is dramatically up-regulated compared to the control (ICV vector) group in PVN (Fig. 1). Co-localization of GFP and DKK1 and up-regulation of DKK1 associated with over-expression of AAV were observed (Fig. 1). In summary, AAV vector successfully mediated over-expression of the target gene DKK1 in PVN.

### Up-regulation of wnt3a and active-β-catenin in PVN in 5/6NX rats was inhibited by central blockade of the wnt signaling

**Fig. 2:**
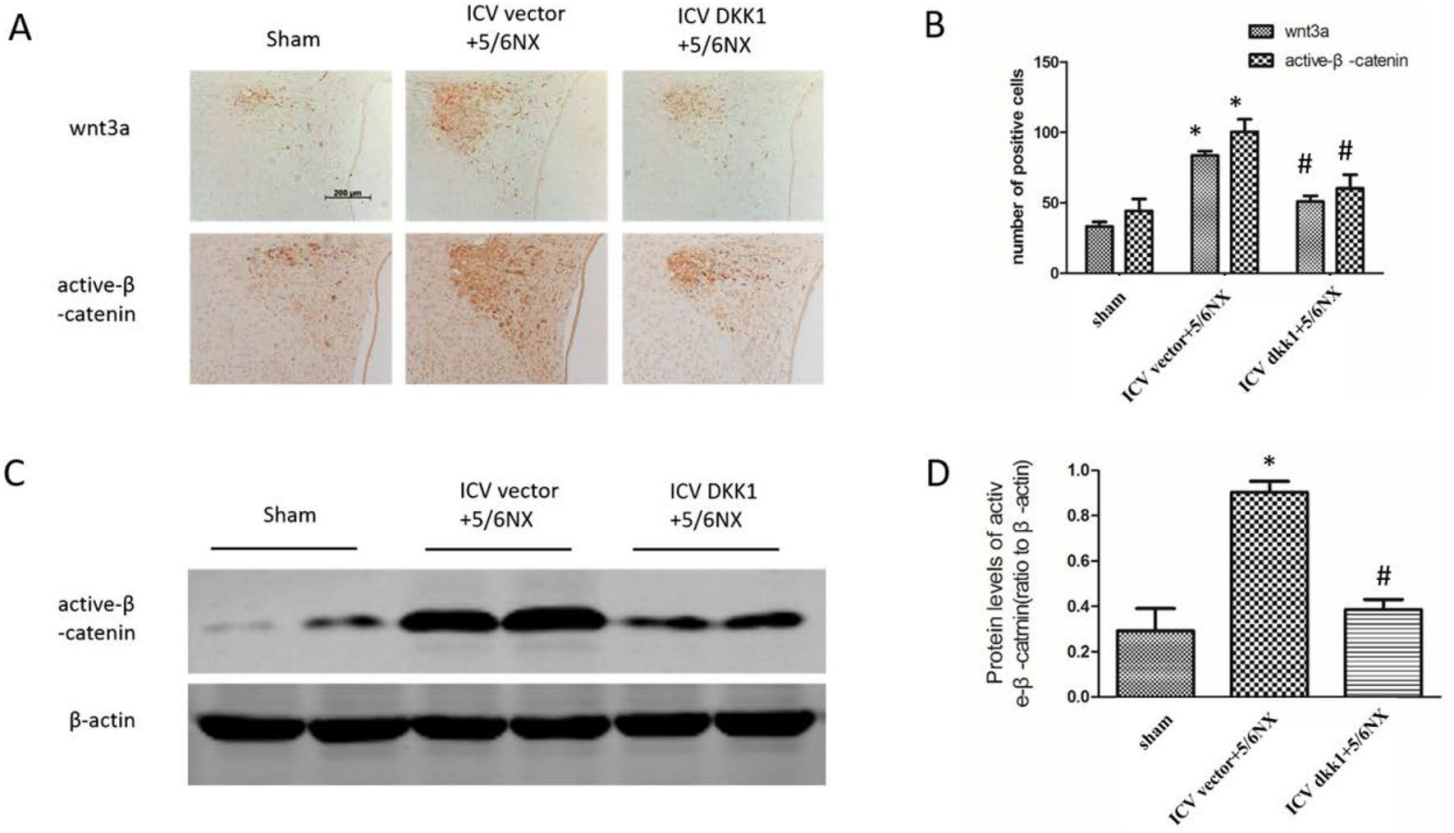
Up-regulation of wnt3a and active-β-catenin in PVN in 5/6NX rats was inhibited by ICV AAV-DKK1. **a** The immunohistochemical images displaying the expression level of wnt3a and active-β-catenin in PVN. **b** The bar graph represent the statistical results of the immunohistochemical analysis. **c** The western blot images displaying the expression level of active-β-catenin in PVN. **d** The bar graph represent the statistical results of the western blot. n = 5 per group, *P < 0.05 vs sham group, ^#^P < 0.05 vs ICV vector+DKK1 group

Wnt/β-catenin signaling plays vital roles in central nervous system, such as development of central nervous system in embryonic period, regulation of nervous system disease and peripheral organ disease. To study the function of wnt/β-catenin signaling in PVN on renal disease, the expression levels of wnt3a and active-β-catenin were assessed by immunohistochemistry and western blot. The results showed that the key molecules of wnt/β-catenin signaling wnt3a and active-β-catenin significantly up-regulated in ICV vector+5/6NX group relative to sham group, but the up-regulation was inhibited in ICV DKK1+5/6NX group (Fig. 2). These results indicated that wnt/β-catenin signaling in PVN was tightly associated to renal disease.

### Up-regulation of RAS components in PVN in 5/6NX rats were inhibited by central blockade of the wnt signaling

**Fig. 3:**
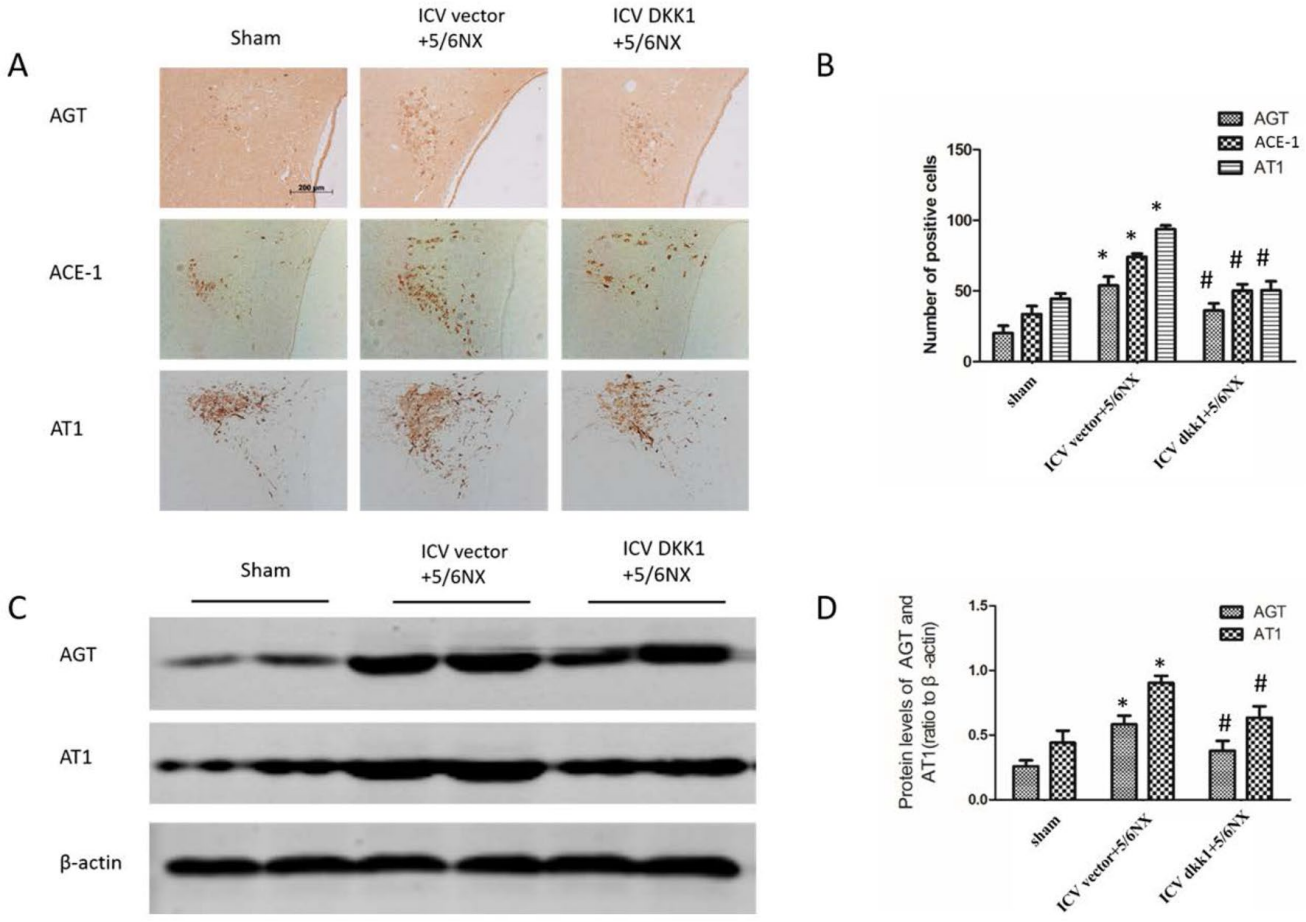
Up-regulation of RAS components, such as AGT, ACE-1 and AT1 in PVN in 5/6NX rats were inhibited by ICV AAV-DKK1. **a** The immunohistochemical images displaying the expression level of AGT, ACE-1 and AT1 in PVN. **b** The bar graph represent the statistical results of the immunohistochemical analysis. **c** The western blot images displays the expression level of AGT and AT1 in PVN. **d** The bar graph represent the statistical results of the western blot. n = 5 per group, *P < 0.05 vs sham group, ^#^P < 0.05 vs ICV vector+DKK1 group

Immunohistochemistry and western blot was performed to assess the expression levels of RAS components including AGT, ACE-1, and AT1. The results showed that AGT, ACE-1, and AT1 significantly up-regulated in PVN in ICV vector+5/6NX group compared with sham group, but the up-regulated expression was inhibited in ICV DKK1+5/6NX group (Fig. 3). It is known that intrarenal RAS plays a vital role in CKD, and is regulated directly by intrarenal wnt/β-catenin signaling. These results suggested that enhanced wnt/β-catenin signaling was upstream and a driving force for RAS activation in brain.

### Deterioration of renal function parameters in 5/6NX rats are dramatically alleviated by central blockade of the wnt signaling

**Fig. 4:**
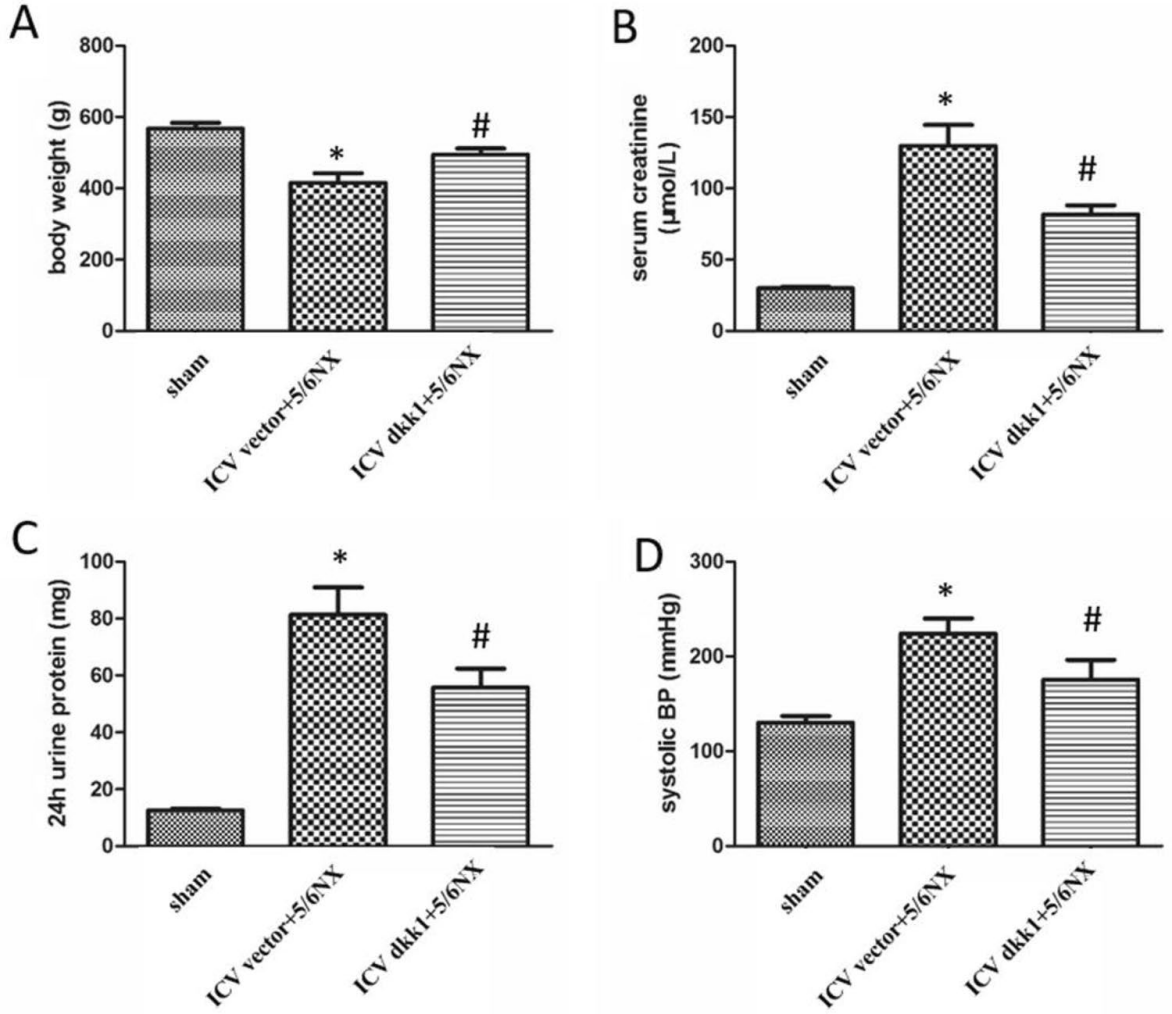
Deterioration of basic parameters, such as systolic blood pressure (BP), serum creatinine, 24 h urinary protein, body weight in 5/6NX rats were dramatically alleviated by ICV AAV-DKK1. **a** The bar graph representing statistical results of bodyweight. **b** The bar graph representing statistical result of serum creatinine. **c** The bar graph representing statistical result of 24 h urine protein. **d** The bar graph representing statistical result of systolic BP. n = 5 per group, *P < 0.05 vs sham group, ^#^P < 0.05 vs ICV vector+DKK1 group

The 5/6NX model was successfully established and showed a progressive rise of systolic blood pressure, serum creatinine, 24 h urinary protein, and terrible loss of weight (Fig. 4). These deteriorative parameters were improved when wnt/β-catenin signaling was selectively blocked by ICV AAV-DKK1. These results proved that central WNT-RAS signaling played a crucial role in progression of chronic kidney disease.

### Over-expression of renal wnt3a and active-β-catenin in 5/6NX rats was inhibited by central blockade of the wnt signaling

**Fig. 5:**
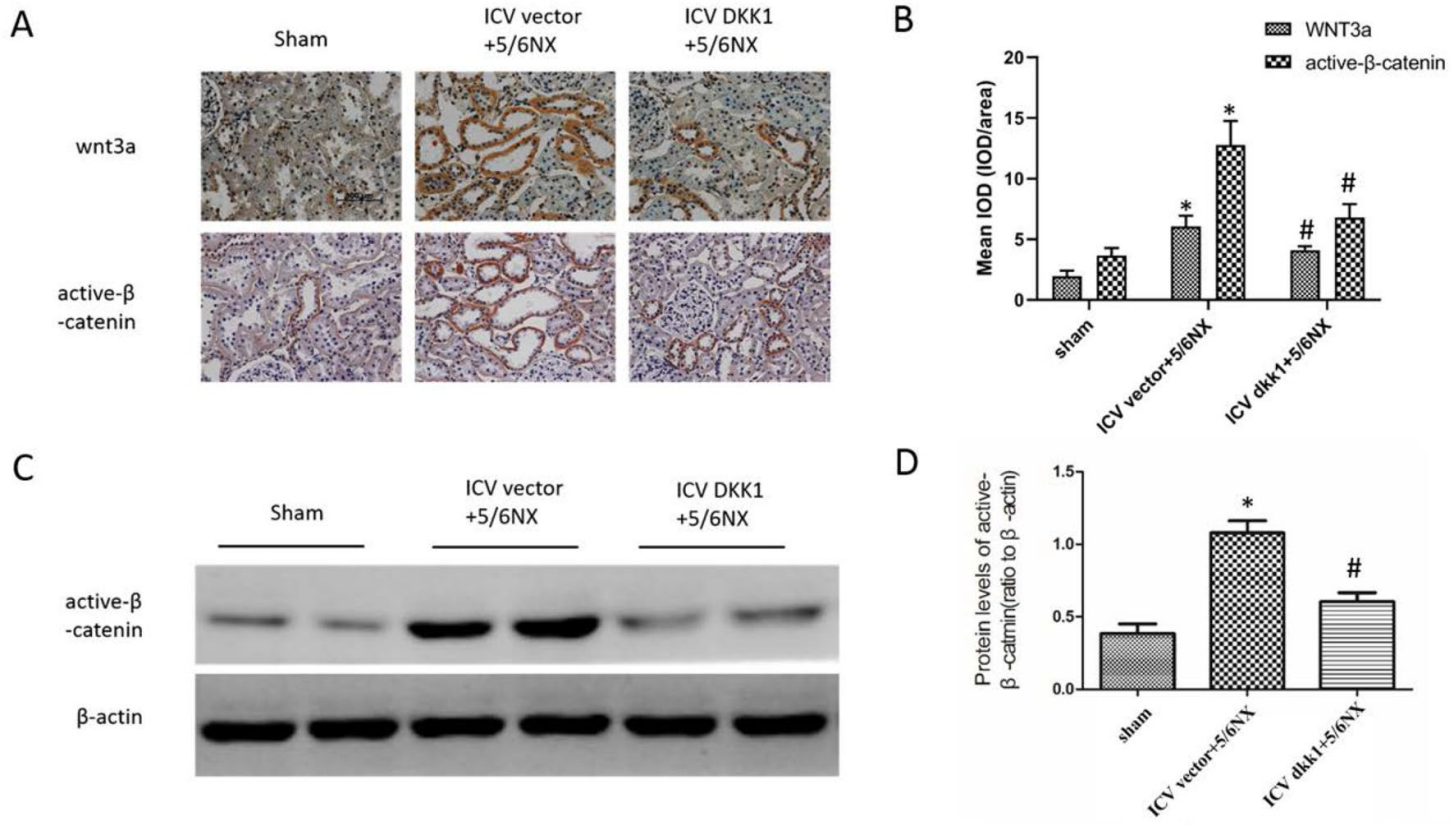
Over-expression of wnt3a and active-β-catenin in kidney in CKD rats was inhibited by ICV AAV-DKK1. **a** The immunohistochemical images displaying the expression level of wnt3a and active-β-catenin in PVN. **b** The bar graph representing the statistical results of the immunohistochemical analysis (10 images/rat). **c** The western blot images displaying the expression level of active-β-catenin in kidney. **d** The bar graph representing the statistical results of the western blot. n = 5 per group, *P < 0.05 vs sham group, ^#^P < 0.05 vs ICV vector + DKK1 group

Expression of intrarenal wnt3a and active-β-catenin, measured by both immunohistochemistry and western blot, was up-regulated significantly after 5/6NX operation, indicating activation of intrarenal wnt/β-catenin signaling in CKD (Fig. 5). Interestingly, over-expression of intrarenal wnt3a and active-β-catenin was down-regulated by central blockade of the wnt signaling by ICV AAV-DKK1 (Fig. 5). These results demonstrated that activation of intrarenal wnt/β-catenin signaling was driven by central wnt/β-catenin signaling.

### Over-expression of intrarenal RAS components in 5/6NX rats was inhibited by central blockade of the wnt signaling

**Fig. 6:**
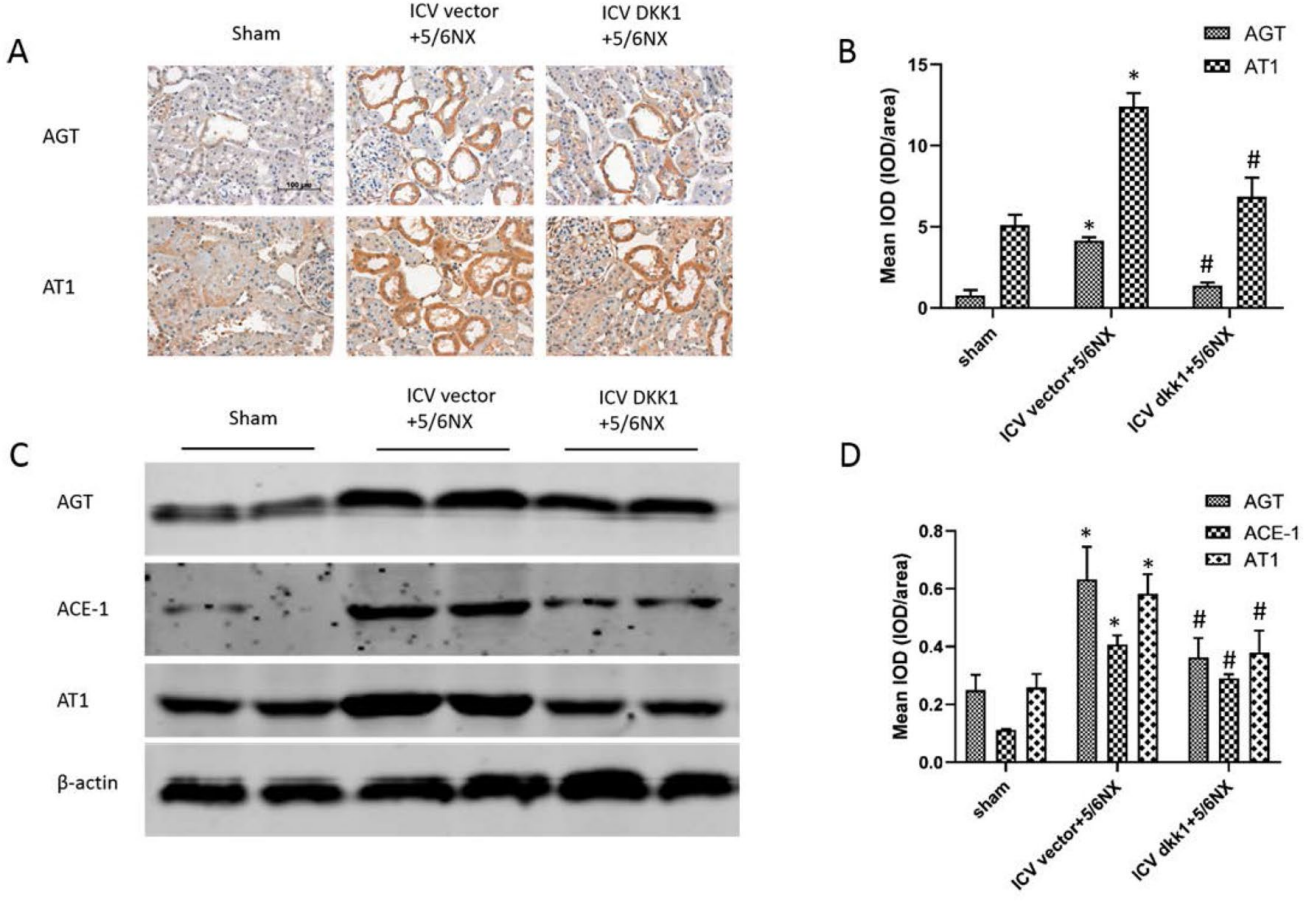
Over-expression of intrarenal RAS components in 5/6NX rats was inhibited by ICV AAV-DKK1. **a** The immunohistochemical images displaying the expression level of AGT and AT1 in PVN. **b** The bar graph representing the statistical results of the immunohistochemical analysis (10 images/rat). **c** The western blot images displaying the expression level of AGT, ACE-1 and AT1 in kidney. **d** The bar graph representing the statistical results of the western blot. n = 5 per group, *P < 0.05 vs sham group, ^#^P < 0.05 vs ICV vector+DKK1 group

Our previous study has revealed that salt-induced strong co-activation and interlinkage of cerebral and intrarenal RAS contributes to renal fibrosis and proteinuria in CKD [17]. Here, the relationship between intrarenal RAS and cerebral wnt/β-catenin signaling was examined through blocking the cerebral wnt/β-catenin signaling by ICV AVV-DKK1. Based on immunohistochemistry and western blot analysis, over-expression of intrarenal RAS components, such as AGT, ACE-1 and AT1 in 5/6NX rats were inhibited by ICV AAV-DKK1 (Fig. 6). These results demonstrated a tight association between central WNT-RAS signaling and intrarenal WNT-RAS signaling.

### Renal fibrosis in 5/6NX rats was significantly attenuated by central blockade of the wnt signaling

**Fig. 7:**
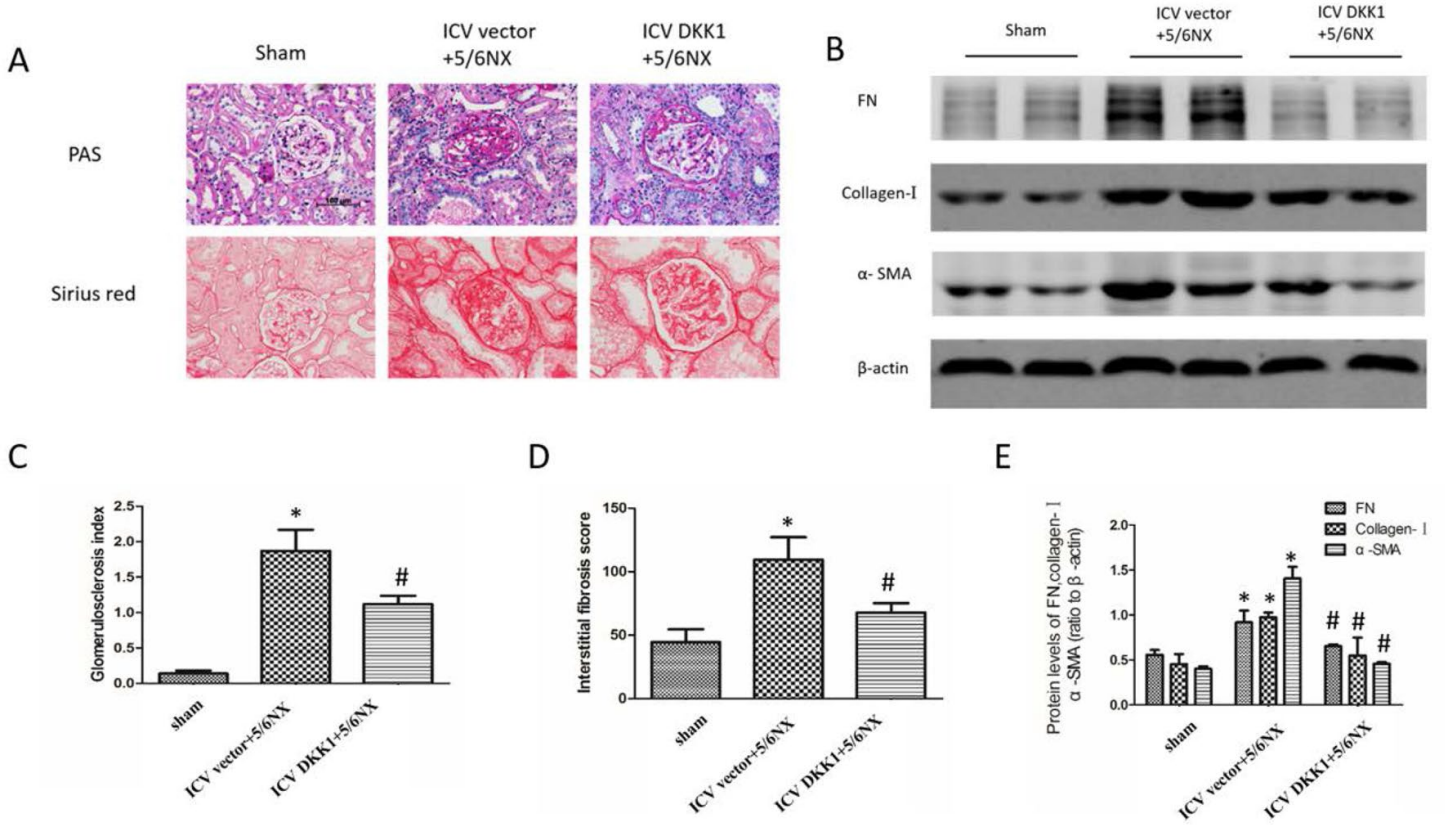
Renal fibrosis in 5/6NX rats were significantly attenuated by ICV AAV-DKK1. **a** The PAS and Sirius red images displaying the level of glomerulosclerosis index and interstitial fibrosis score in kidney, respectively. **b** The western blot images displaying the expression level of Fibronectin (FN), Collagen-I and α-SMA in kidney. **c**, **d** Bar graphs representing the statistical results of the PAS (**c**) and Sirius red (**d**), respectively (20 images/rat). **e** The bar graph representing the statistical results of the western blot in (B). n = 5 per group, *P < 0.05 vs sham group, ^#^P < 0.05 vs ICV vector+DKK1 group

The pathologic changes of kidney tissue in 5/6NX rats were observed by PAS and Sirius red staining. The glomerulosclerosis index was significantly higher after 5/6NX. Central blockade of the wnt signaling by ICV AAV-DKK1 significantly improved glomerulosclerosis in 5/6NX rats detected by PAS (Fig. 7a, c). In addition, interstitial fibrosis in 5/6NX rats was attenuated by central blockade of the wnt signaling by ICV AAV-DKK1 as assessed by Sirius red staining (Fig. 7a, d). As illustrated in Fig. 7b, e), the over-expression of profibrotic molecules, such as Fibronectin (FN), Collagen-I, and α-SMA was observed in kidney homogenates of 5/6NX rats as detected by western blot. After treatment of ICV AAV-DKK1, the upregulated FN, collagen-I and α-SMA protein levels were significantly inhibited. These results indicated that the development and progression of renal fibrosis were regulated by cerebral WNT-RAS signaling.

## Discussion

**Fig. 8:**
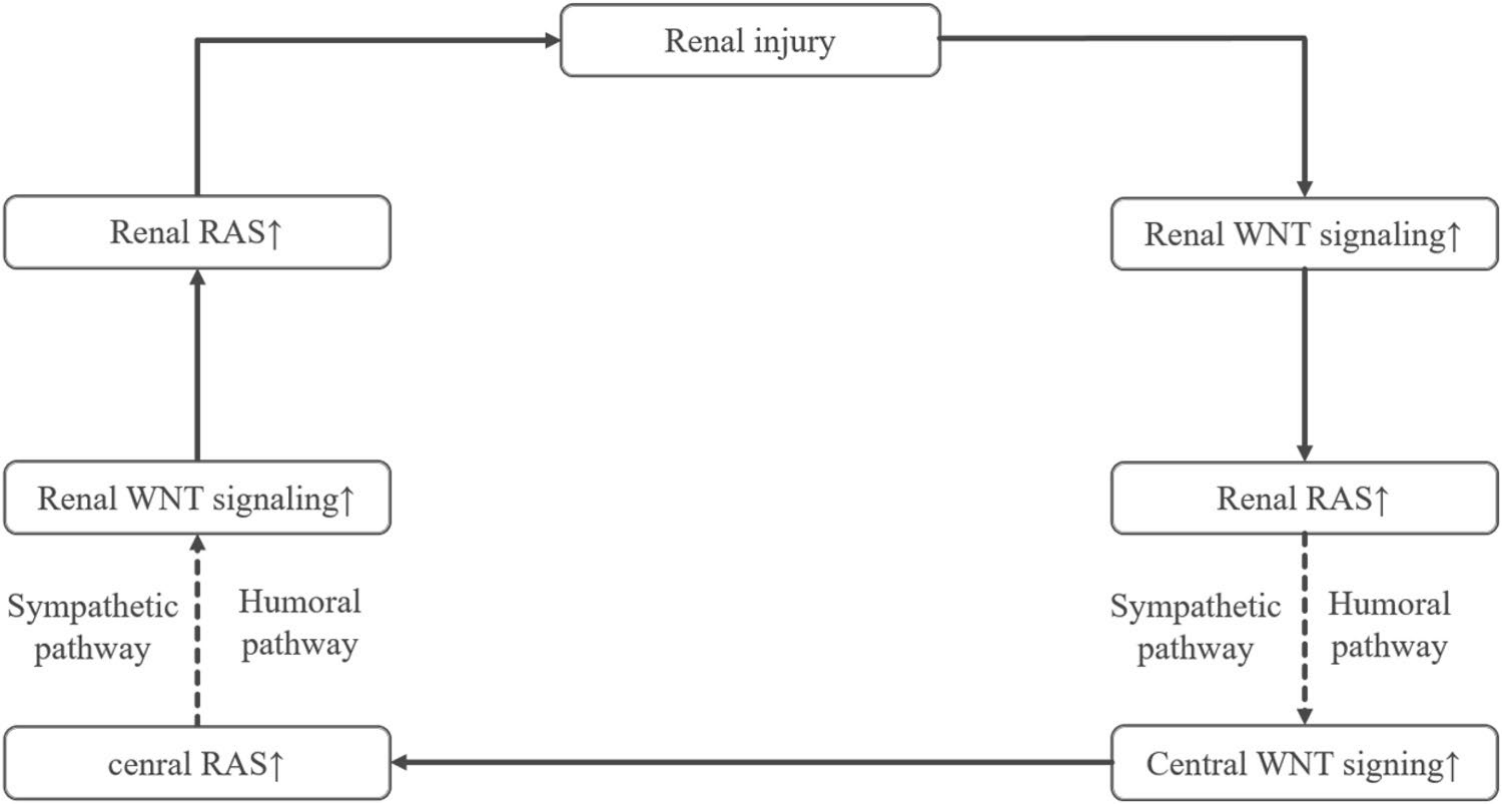
Schematic diagram summarizing coactivation of central and renal WNT-RAS signaling in the damaged kidney

Abnormal activation of wnt/β-catenin signaling and RAS is known to play a vital role in the development and progression of CKD. It has been well recognized that the PVN is a vital integrative nucleus that expresses RAS and can markedly influence blood pressure and sympathetic nerve activity [17, 19]. In this study, we found that cerebral and intrarenal WNT-RAS signaling was activated in 5/6NX rats. More importantly, blockade of cerebral wnt signaling significantly improved interstitial fibrosis and renal function. Central and intrarenal WNT-RAS signaling forms a vicious circle deteriorating renal injury, which is illustrated in Fig. 8. This study revealed the important role of central WNT-RAS signaling in the development and progression of CKD and provided a novel pathological mechanism of CKD.

The functions of central nervous system on renal disease have been reported, and the pathway of signal communication between central nervous system and kidney has been demonstrated [17, 19]. The PVN is a vital cardiovascular center which expresses major RAS components, such as AGT, ACE-1, AT1, and contributes to hypertension in CKD [21–23]. Wnt/β-catenin signaling is known to be involved in embryonic development, tissue homeostasis as well as organ injury and repairment [1–3]. Dysregulation of the wnt/β-catenin signaling leads to multiple diseases, such as hereditary diseases, colon cancer and renal fibrosis [4–6]. A growing body of research has reported that wnt/β-catenin signaling plays important roles in renal diseases, include acute and chronic renal diseases, repairing function or damaging function [5, 7, 8]. The roles of wnt/β-catenin signaling in renal fibrosis have attracted wide attention. In this study, we found that cerebral and intrarenal wnt/β-catenin signaling components, such as wnt3a and active-β-catenin, were up-regulated in 5/6NX rats and the over-expression was repressed by ICV AAV-DKK1. DKK1, a wnt antagonist, inhibits activation of wnt/β-catenin signaling pathway by competing for the shared receptor LRP5/6 and consequently inhibits RAS activation.

We have previously reported that the RAS signaling in PVN plays a significant influence to renal fibrosis [17, 19]. In this study we have found that cerebral and intrarenal RAS components, such as AGT, ACE-1 and AT1, were up-regulated in 5/6NX rats and the over-expression was repressed by central blockade of the wnt signaling and consequently RAS activation was inhibited. These findings demonstrated that RAS is regulated by wnt/β-catenin signaling not only in kidney, but also in brain. RAS play a key role in the development and progression of CKD, and the inhibitors of RAS, such as ACEI and ARB, are the mainstays of current clinic therapy for CKD [15, 16]. ACEI inhibits the catalytic activity of ACE and blocks conversion of angiotensin I to Ang II, the major pathogenic culprit of RAS [11–14]. Therefore, our finding may provide a theoretical foundation for treatment of CKD, and provide a new possibility to solve the side effect of RAS inhibitor, such as compensatory expression of renin following anti-RAS therapy, since inhibitor of wnt signaling which can affect almost all of RAS component, is different to conventional RAS blockers.

Interestingly, blockade of central wnt signaling by ICV AAV-DKK1 dramatically attenuated the renal pathological changes in 5/6NX rats, including glomerulosclerosis and interstitial fibrosis detected by PAS and Sirius red staining. Simultaneously, the expression of FN, collagen-I and α-SMA, the major ingredients of extracellular matrix, was evaluated by western blot, and the results showed that over-expression of these proteins in CKD were obviously alleviated by ICV AAV-DKK1. These data clearly showed that the development and progression of renal fibrosis were regulated by central WNT-RAS signaling. This is the first report illustrating that central WNT-RAS signaling makes a vital difference to renal fibrosis.

Renal fibrosis is a common pathway of virtually all progressive kidney diseases in CKD patients and the major factor influencing prognosis [24, 25]. There is no doubt that these findings will offer a new idea to treat or slow down the progression of CKD. However, some issues remain to be studied. Wnt family include many kinds of wnt molecules, such as wnt1, wnt3a, wnt4, wnt7 and so on. The potential roles of other wnt molecules in brain except wnt3a in renal diseases remain unknown [3]. In addition, the mechanism underlying Reno-Cerebral communication has not been demonstrated in this study. However, previous studies have established the related mechanisms: (1) Sympathetic afferent and efferent nerves serve as a bridge between kidney and brain, and (2) Humoral signaling molecules directly binds to the receptors distributed in the nucleus which lacks blood–brain barrier and results in a series of changes [17, 19, 23].
